# Antigenic and structural analysis of the influenza hemagglutinin lateral patch

**DOI:** 10.1128/jvi.01265-26

**Published:** 2026-09-23

**Authors:** Jordan I. Lemus-Reyes, Monica L. Fernández-Quintero, Edgardo Ayala, Olivia M. Swanson, Marina R. Good, Wei Ji, Devika Suja, Julianna Han, Andrew B. Ward, Jenna J. Guthmiller

**Affiliations:** 1 Department of Immunology and Microbiology, University of Colorado Anschutz Medical Campus 129263, Aurora, Colorado, USA; 2 Department of Integrative Structural and Computational Biology, The Scripps Research Institute 4356, La Jolla, California, USA; St Jude Children's Research Hospital, Memphis, Tennessee, USA

**Keywords:** H1N1, monoclonal antibodies, antigenic drift, influenza virus

## Abstract

The lateral patch epitope of the H1 hemagglutinin (HA) was a dominant target of antibodies following exposure to the 2009 pandemic H1N1 virus. However, the conservation and mechanisms of antigenic drift in the lateral patch remain unresolved. Here, we used lateral patch-specific monoclonal antibodies (mAbs) to understand the antigenicity of the lateral patch of human, avian, and swine H1N*x* viruses spanning from 1918 to 2022. We found that lateral patch-specific mAbs demonstrated broad binding to pre-2009 H1N1 viruses and to the pandemic 2009 H1N1. However, we identified discrete mutations that drove multiple antibody evasion pathways for lateral patch-targeting mAbs in post-2009 H1N1 viruses. Despite this, we observed that the lateral patch remains well conserved across enzootic sources, suggesting that existing lateral patch antibodies could protect against a future zoonotic H1N*x* pandemic. Together, these data support that the lateral patch remains an attractive target for pandemic preparedness against emergent H1N*x* viruses.

**IMPORTANCE:**

Seasonal influenza viruses continually undergo antigenic drift to evade antibody-mediated immunity, yet how individual antibody specificities shape this evolution remains poorly understood. We investigated the conservation and antigenic evolution of the H1 hemagglutinin (HA) lateral patch, an epitope targeted by broadly neutralizing antibodies. Using human monoclonal antibodies, we found that the lateral patch had remained highly conserved across nearly a century of human and animal influenza virus circulation but has undergone antigenic drift multiple times in pre- and post-2009 seasonal H1N1 viruses. Structural and mutational analyses revealed how these substitutions alter antibody recognition and shape the genetic features of lateral patch-targeting antibodies across individuals born in different eras. The lateral patch is also conserved among swine and avian H1N*x* viruses, highlighting its potential as a target of protective immunity against pandemic H1N*x* viruses. These findings provide a comprehensive framework for understanding antibody-driven evolution within a broadly conserved influenza virus epitope.

## INTRODUCTION

Influenza A viruses (IAVs) are one of the most common infectious respiratory diseases that threaten the human population. Estimates of seasonal influenza-associated mortality range from 290,000 to 645,000 deaths annually, and more than 5 million influenza-associated hospitalizations occur each year (1, 2). Beyond seasonal epidemics, IAVs remain a tangible pandemic threat, given their propensity for interspecies transmission, infecting a broad range of avian and mammalian hosts, including swine (3, 4). This broad host range can lead to enzootic spillover events that generate reassortant viruses capable of infecting multiple species. These IAV spillovers to humans have led to four pandemics, two of which were caused by the H1N1 subtype in 1918 and 2009 of avian and swine origins, respectively (5–7). Current efforts to curb the endemic spread of seasonal IAV subtypes, H1N1 and H3N2, are largely focused on annual vaccination efforts. However, influenza viruses rapidly acquire mutations, a process known as antigenic drift, to evade neutralizing antibody responses elicited by past natural infections or vaccinations, prompting the annual reformulation of the influenza vaccine (8).

Antibody responses primarily target the globular head domain of the surface glycoprotein hemagglutinin (HA) (9). The HA of H1N1 has five major antigenic sites (Ca1, Ca2, Cb, Sa, and Sb) that are the main targets of head-specific antibodies induced by infection and vaccination (9). Antibodies targeting the major antigenic sites typically have narrow binding breadth, limiting their ability to neutralize antigenically drifted viruses. Despite this antigenic variability, potent and broadly neutralizing antibodies can target conserved epitopes on the HA head domain, specifically the receptor-binding site (RBS) and the lateral patch (10–14). The RBS directly interacts with the host receptor, sialic acid, and thus needs to be conserved to maintain optimal receptor binding. In contrast, the lateral patch is found on the backside of the HA protomer and is largely composed of the 160-loop of major antigenic site Sa, 170-loop of Ca1, and the 120 β-strand (11, 13). The function of the lateral patch is currently unknown. Previous work has demonstrated that humans induce robust antibody responses against the lateral patch following vaccination with the 2009 pandemic H1N1 (pH1N1) virus, with lateral patch-specific antibodies able to neutralize pre- and post-2009 H1N1 viruses and mediate protection against pH1N1 *in vivo* (11, 14, 15). However, a focused antibody response against the lateral patch led to antigenic drift during the 2013–2014 influenza season (14, 15), resulting in a seasonal influenza virus outbreak with greater morbidity and mortality than the 2009 pandemic, particularly among middle-aged adults (16, 17). Despite its rapid antigenic drift, the lateral patch epitope remained well conserved between 1977 and 2009 (11–15). The conservation of the lateral patch across time and animal reservoirs remains ill-defined.

Here, we utilized lateral patch-targeting monoclonal antibodies (mAbs) isolated from humans to assess the antigenicity of the lateral patch across historical H1N*x* isolates from humans, swine, and avian species. We assessed lateral patch-specific mAb binding affinity to H1 HA antigens from 1918 to 2022 to understand the conservation of the lateral patch in seasonal H1N1 (sH1N1, 1918–1957 and 1977–2009), pH1N1 (2009–present), and enzootic H1N*x* isolates. Lateral patch-specific mAbs used in this study were originally identified in prior studies (11, 13). In this study, we have classified lateral patch-specific mAbs into two classes based on their antibody gene usage and angle of approach to the lateral patch. Moreover, these antibody classes were linked to donor age; class 1 lateral patch-specific mAbs were found in individuals born post-1980, whereas class 2 lateral patch-specific mAbs were found in individuals born before 1977. By testing these discrete mAb classes against a range of pH1N1 virus recombinant HAs (rHAs), we identified critical mutations in HA that have emerged and persisted in post-2009 pH1N1 that readily evade both mAb classes. Despite this, we found one lateral patch-specific mAb that maintains binding to current pH1N1 viruses, suggesting the lateral patch is still an attractive target for generating humoral immune responses. Lateral patch-specific mAbs demonstrated broad reactivity to sH1N1 and to the 2009 pH1N1 virus. However, we identified mutations in sH1N1 in the late 1950s and the late 1970s when sH1N1 reemerged that specifically ablated class 1 lateral patch-specific mAb binding, which could explain the birth-year-specific patterns observed in the distinct lateral patch-binding mAb classes. Finally, we found that most lateral patch-targeting mAbs bind to animal-origin H1N*x* antigens, suggesting that the lateral patch epitope is well conserved and could be targeted to mediate protection in the event of zoonotic H1N*x* spillovers. Together, these data support that the lateral patch remains an attractive target for eliciting antibody responses to mediate protection against pandemic-potential H1N*x* viruses.

## RESULTS

### Evolution of H1 HA across time and species

**Fig 1:**
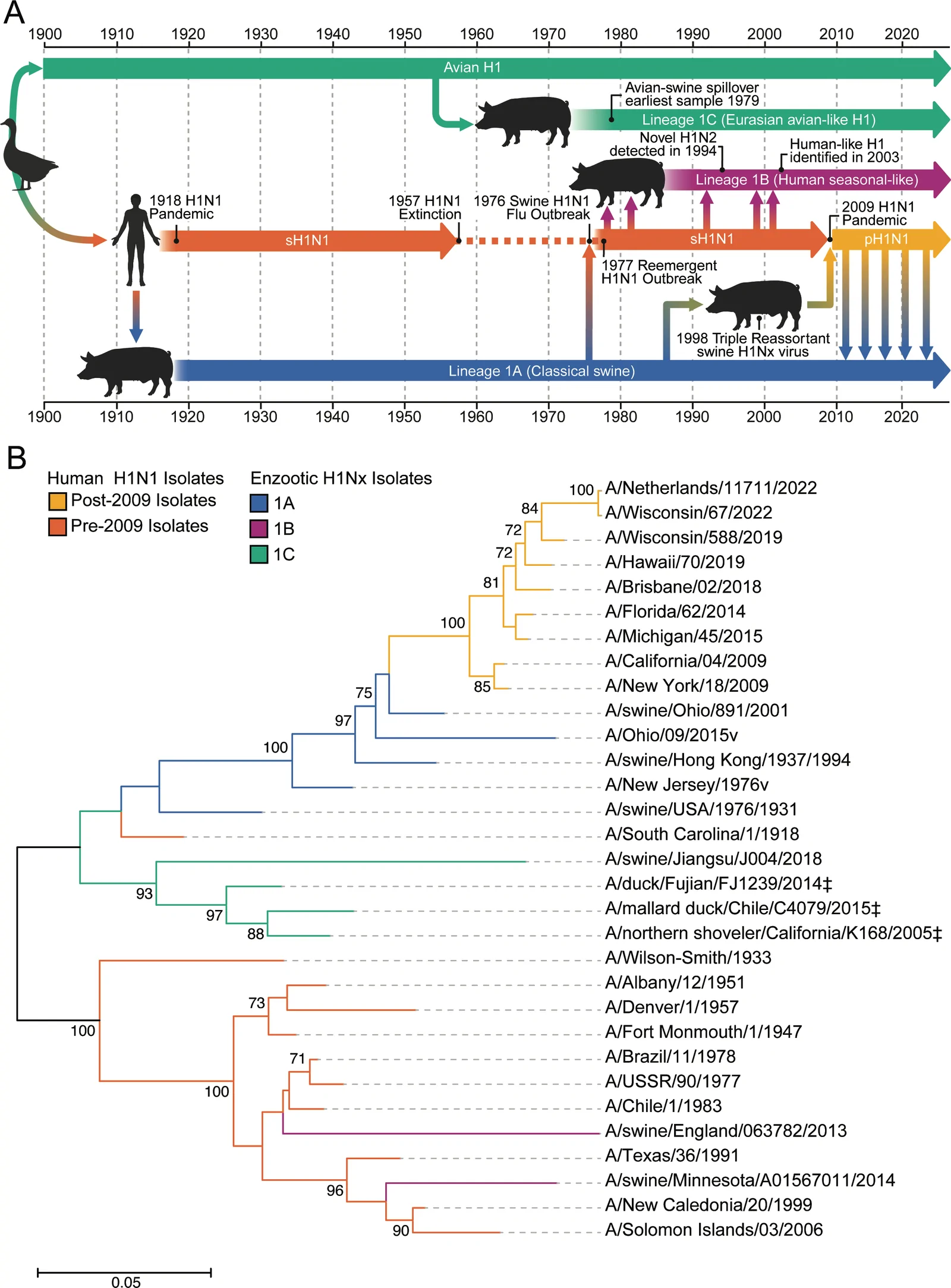
Genetic and phylogenetic relationship of the H1 HA influenza virus in human and zoonotic isolates based on the amino acid sequence. (**A**) Timeline of human, swine, and avian H1 lineages. (**B**) Analysis of 31 HA sequences based on the best neighbor-joining method was used to analyze 31 HA sequences from this study (see Table S1). Branch colors are used to distinguish pre- and post-2009 human H1N1 isolates and zoonotic isolates across lineages. The scale bar indicates the branch length corresponding to 0.050 amino acid substitutions per site using the Jones-Taylor-Thornton (matrix-based method). ‡Avian H1s are marked as 1C lineage, as no clade classification system exists for avian H1N*x* viruses.

To understand the conservation and antigenic drift of the lateral patch, we assessed lateral patch antibody binding to recombinant HA (rHA) antigens from a wide range of H1N*x* viruses. H1N1 viruses have circulated in humans since the 1918 pandemic, with a brief gap from 1957 to 1977, with the original 1918 virus deriving from an ancestral avian H1N1 virus (18) (Fig. 1A). The 1918-lineage seasonal H1N1 (sH1N1) circulated from 1918 to 1957, before being replaced in 1957 by H2N2. sH1N1 viruses resembling those from the 1950s reemerged in 1977 and persisted in humans until 2009 (19, 20). sH1N1 viruses went extinct in 2009 due to the emergence of the 2009 pH1N1 virus, which derived its HA from the classical swine lineage 1A (7). In parallel, swine H1N*x* viruses have remained in circulation since the 1918 pandemic and have caused occasional swine-to-human influenza infections, including the swine-origin H1N1 influenza outbreak at Fort Dix, NJ, USA, in 1976 (21). A new triple reassortant virus emerged, generating the H1N1 swine virus, which ultimately led to the 2009 pH1N1 pandemic (22). Drifted variants of pH1N1 continue to circulate in humans and cause seasonal outbreaks.

H1N*x* viruses circulate in avian and swine hosts, contemporaneously with H1N1 viruses that circulate in humans (Fig. 1A), with three swine lineages deriving from distinct human and avian origins (23). The 1A classical swine lineage is closely related to the 1918 H1N1 pandemic virus and circulates globally (18), particularly subclade 1A.3.3.2 (24). This subclade was the result of reverse zoonosis of the 2009 pH1N1 lineage in pigs, a frequent event that still occurs with contemporary pH1N1 viruses (25–27). Similarly, the 1B swine lineage is derived from multiple, separate events of reverse zoonosis of human sH1N1 viruses between 1977 and 2009. The first 1B clade spillover, initially detected in 1994 in Great Britain and later spreading throughout Europe, likely originated from a circulating sH1N1 virus in the early 1980s (28, 29). Two separate introductions of sH1N1 into swine in the early 2000s were first detected in the US in 2005 (30–32) (Fig. 1A and B). These independent spillovers correspond to two distinct 1B clades, each associated with different geographical regions: 1B.1 H1N*x* is most prevalent in the United Kingdom and Europe (23, 28, 33), whereas 1B.2 H1N*x* predominantly circulates in the Americas and Asia (23, 31). Despite these two distinct clades, there is phylogenetic evidence suggesting human seasonal-like H1N1 viruses infected swine populations as early as the 1970s, 1990s, and 2000s in separate geographical regions (34). The 1C swine lineage, or the Eurasian avian (EA)-like H1 lineage, is derived from avian H1N*x* viruses and entered the swine population in the 1970s, first appearing in Europe and subsequently spreading throughout the Eurasian continents (30, 35) (Fig. 1A). Avian H1s used in this study are classified as 1C lineage, given their similarity to swine 1C on sequence-based analysis and because no clade classification system exists for circulating avian H1N*x* viruses (23, 36) (Fig. 1B).

We generated a phylogenetic tree of H1 amino acid sequences derived from sH1N1, pH1N1, the three H1 swine lineages, and the avian H1s related to swine 1C viruses to show the relationship between human and enzootic H1s. The joint-joining phylogenetic tree was inferred using all representative H1 HA amino acid sequences (Table S1) used to screen lateral patch-specific mAbs to reveal the sequence diversity of known circulating isolates collected over time, from differing host origins, and across various geographical regions. Lineage and clade designations are shown alongside their corresponding branch colors (Fig. 1A and B). 1A and 1B strains cluster near human isolates, indicating the role of reverse zoonosis in their origin. Isolates such as A/New Jersey/1976v (NJ/76v) and A/Ohio/09/2015v (OH/15v) are human variant isolates that result from swine-to-human spillovers and are atypical of human seasonal influenza viruses. The HA amino acid segment showed bootstrap support of 70% or more in 1,000 replicates, as indicated by the nodes, particularly in most human isolates and a few enzootic isolates (Fig. 1B). The A/South Carolina/1/1918 (SC/18) virus established the classical swine lineage (1A), which subsequently gave rise to the pH1N1 viruses. These data support that the reverse zoonosis of a 1918-like H1N1 virus in pigs drifted slowly due to the close phylogenetic relatedness of these strains. SC/18 was also distinct from other post-1918 sH1N1 viruses, which exhibit substantial amino acid diversity from the 1918 isolate. However, the accuracy of older strain sequences (1930s–1950s) remains uncertain, as these viruses were serially passaged in embryonated chicken eggs prior to sequencing, introducing mutations not observed in circulating strains (37, 38). However, it is possible that the mutations gained were due to antigenic drift and reverted back in later isolates. sH1N1 viruses continued to undergo antigenic drift until they went extinct in 2009 due to the emergence of the pH1N1 virus.

Despite their genetic differences, enzootic isolates have limited genetic diversity, specifically in lineages 1A and 1C. This lack of diversity could suggest these viruses undergo slower antigenic drift relative to human H1N1 viruses, potentially as the longevity of these animals’ lifespan limits the immune pressure placed on enzootic-origin H1N*x* viruses (39, 40) (Fig. 1B). However, the increase in genetic and antigenic diversity of H1s is caused by intensive farming practices, including indoor housing, transportation within and between farms, naïve and herd-level immunity, and interactions between various species of IAV-susceptible hosts (4, 41). While there may have been a greater divergence of amino acid substitutions acquired over time in enzootic H1s (Fig. 1B), this may be attributed to substantiated gaps in enzootic H1N*x* surveillance (42). In addition, genomic surveillance and tracking of swine IAVs are limited and challenging due to limited resources among geographical regions (43, 44). Together, these data support three major branches of H1 diversity related to the initial 1918 spillover into humans and the classical swine lineage (1A), viruses deriving from humans to swine from 1977 to 2009 (1B), and the avian and EA-like swine lineages of H1 (1C).

### Rapid antigenic drift in the lateral patch of post-2009 H1N1 viruses

**Fig 2:**
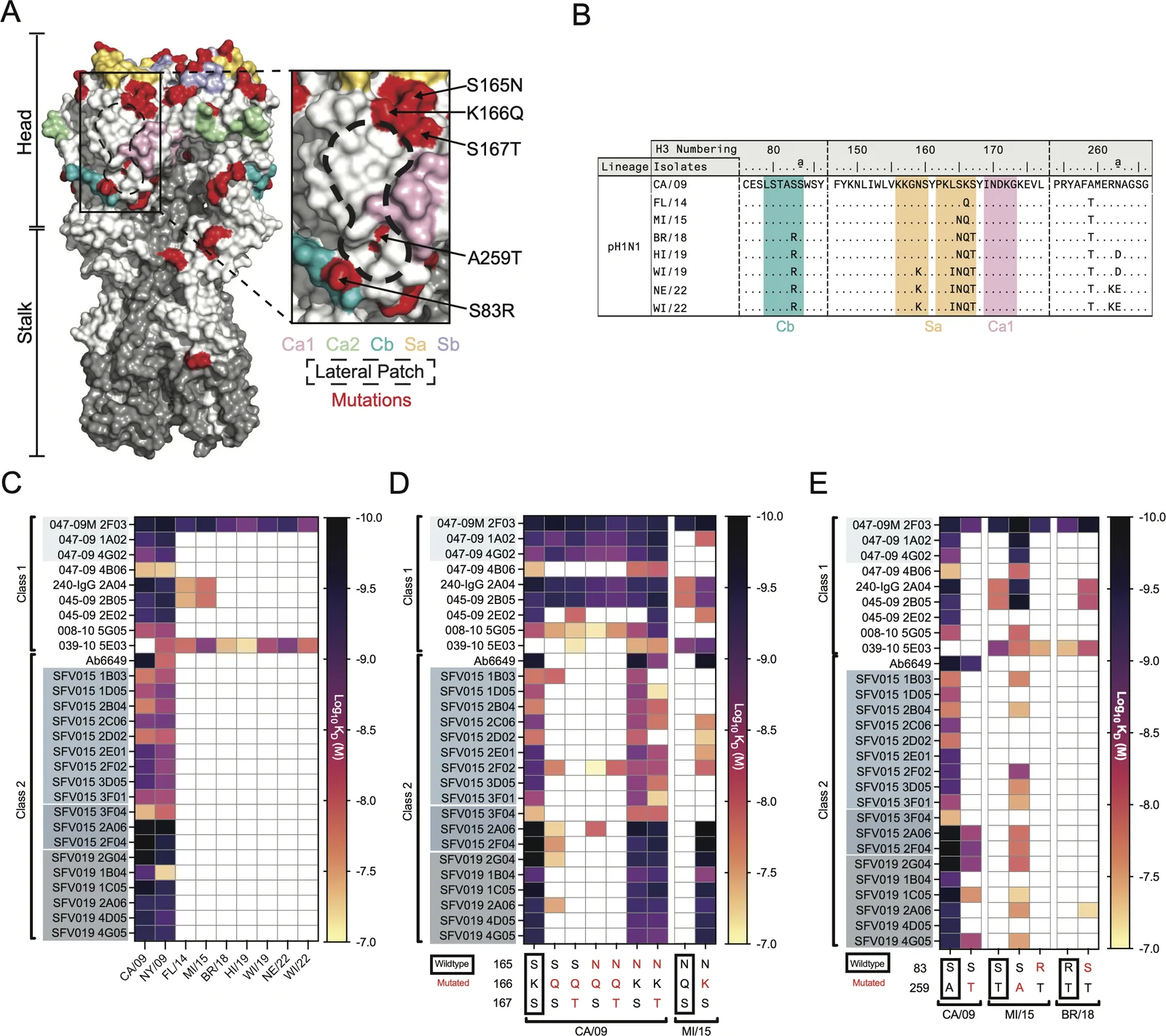
Antigenic structure and binding breadth of lateral patch-specific mAbs to post-2009 H1N1 viruses. (**A**) Structure of HA from A/California/04/2009 (PDB: 4JTV), highlighting the major antigenic sites (Ca1, Ca2, Cb, Sa, and Sb), the lateral patch epitope, and mutations in post-2009 pH1N1 viruses. The HA1 and HA2 are visualized in white and gray, respectively. (**B**) Amino acid sequence alignment of post-2009 H1 isolates of residues within or near the lateral patch. (**C–E**) Apparent affinity of lateral patch-targeting mAbs against post-2009 rH1 isolates. mAbs are divided by their class. Clonally related mAbs are highlighted by distinctly shaded blue boxes. (**D and E**) rH1 isolates with different mutations at positions 165/166/167 (**D**) and at 83 and 259 (**E**). White boxes represent no binding.

Since their introduction in 2009, pH1N1 viruses have undergone significant antigenic drift (45, 46). Between 2010 and 2020, 29 amino acid substitutions emerged within the HA head domain (Table S2); 11 mutations occurred within the variable antigenic sites (Ca1, Ca2, Cb, Sa, and Sb), with 1 mutation introducing an N-glycosylation motif at position 165 (Fig. 2A). The lateral patch epitope lies in between the Ca1, Cb, and Sa antigenic sites, where a total of five mutations were acquired in and around the lateral patch in drifted pH1N1 viruses (Fig. 2A and B).

To understand how antigenic drift in post-2009 H1N1 viruses has affected the antigenicity of the lateral patch, we evaluated the binding breadth and affinity of lateral patch-targeting mAbs against rHAs from post-2009 H1N1 viruses (Fig. S1). mAbs used in this study were largely generated from B cells isolated from human cohorts following the 2009 pH1N1 monovalent influenza vaccine or the 2014–2015 quadrivalent vaccine (Table S3) (11, 47, 48). We also analyzed the antigenicity of the lateral patch using Ab6649, the first characterized mAb targeting the lateral patch (13). Lateral patch-targeting mAbs utilize highly restricted variable heavy (V_H_) and variable kappa (V_K_) genes (11). We have grouped lateral patch-specific mAbs into two classes based on their V_H_ genes (Table S) and their angle of approach (11). Class 1 mAbs utilize V_H_3-23, commonly paired with V_K_1-33. In contrast, Class 2 mAbs largely used V_H_ 1-2, V_H_ 3-74, or V_H_ 3-7 paired with V_K_1-33.

We found that both class 1 and class 2 antibodies lost binding to pH1N1 strains around 2014, suggesting antigenic drift affected mAb binding (Fig. 2C). In 2012, pH1N1 viruses acquired K166Q, located in the Sa antigenic site (Fig. 2A), which became dominant in the 2013–2014 influenza season and has persisted in circulating pH1N1 viruses (Fig. 2B). Moreover, two additional mutations occurred directly before (S165N) and after (S167T) the K166Q mutation (Fig. 2B). These mutations occur in A/Michigan/45/2015 (MI/15; S165N and K166Q) and A/Brisbane/02/2018 (BR/18; S165N, K166Q, and S167T). To dissect the individual roles of these three mutations on mAb binding, we generated rHAs with various combinations of mutations at positions 165–167. We found K166Q alone dramatically reduced binding of class 2 mAbs, whereas S165N and S167T had little to no effect on binding in mutants on the A/California/04/2009 (CA/09) background (Fig. 2D). The crystal structure of mAb Ab6649 in complex with a 2006 H1 revealed that this antibody directly binds to K166 (13) and is classified as class 2 despite containing a different antibody gene repertoire (Table S3). The class 2 mAb 6649 was likely susceptible to antigenic drift at this site (13). This finding, as well as evidence that the class 2 mAb Ab6649 binding footprint overlaps with K166, could provide an explanation as to why class 2 mAbs are more susceptible to the K166Q mutation. Reversion to Q166K in the MI/15 background restored binding by most class 2 lateral patch antibodies and, surprisingly, some class 1 mAbs (Fig. 2D). Together, these data support that direct interaction of class 2 lateral patch-specific mAbs with K166 could be responsible for the loss of binding when the K166Q mutation emerged, in agreement with several other studies (11, 13–15).

A259T and K166Q occurred in post-2009 pH1N1 viruses, with both mutations found near the lateral patch epitope (Fig. 2A). A259T emerged around 2012 and is ubiquitous in circulating pH1N1 viruses (Fig. 2B; Table S2). We introduced this substitution in CA/09 and found the majority of class 1 mAbs and a subset of class 2 mAbs lost binding (Fig. 2E). Reversion to T259A in MI/15 led to the restoration of binding by most class 1 lateral patch mAbs. Surprisingly, many class 2 mAbs regained binding to the T259A MI/15 despite the presence of the K166Q mutation, albeit with lower binding affinity than WT CA/09 (Fig. 2E). These data suggest that only two mutations, K166Q and A259T, work in concert to evade the vast majority of lateral patch mAbs.

Lastly, S83R mutation emerged in the Cb antigenic site in post-2015 pH1N1 isolates and is present in the BR/18 pH1N1 isolate (Fig. 2E; Table S2). Reversion of this mutation in the BR/18 background or substitution in MI/15 revealed this mutation was necessary and sufficient for evasion of 240-IgG 2A04 and 045-09 2B05 (Fig. 2E). Together, these data support that multiple distinct mutations in and around the lateral patch of post-2009 pH1N1 viruses have emerged to evade lateral patch-specific antibodies.

#### Structural analysis of class 1 lateral patch-specific mAbs

**Fig 3:**
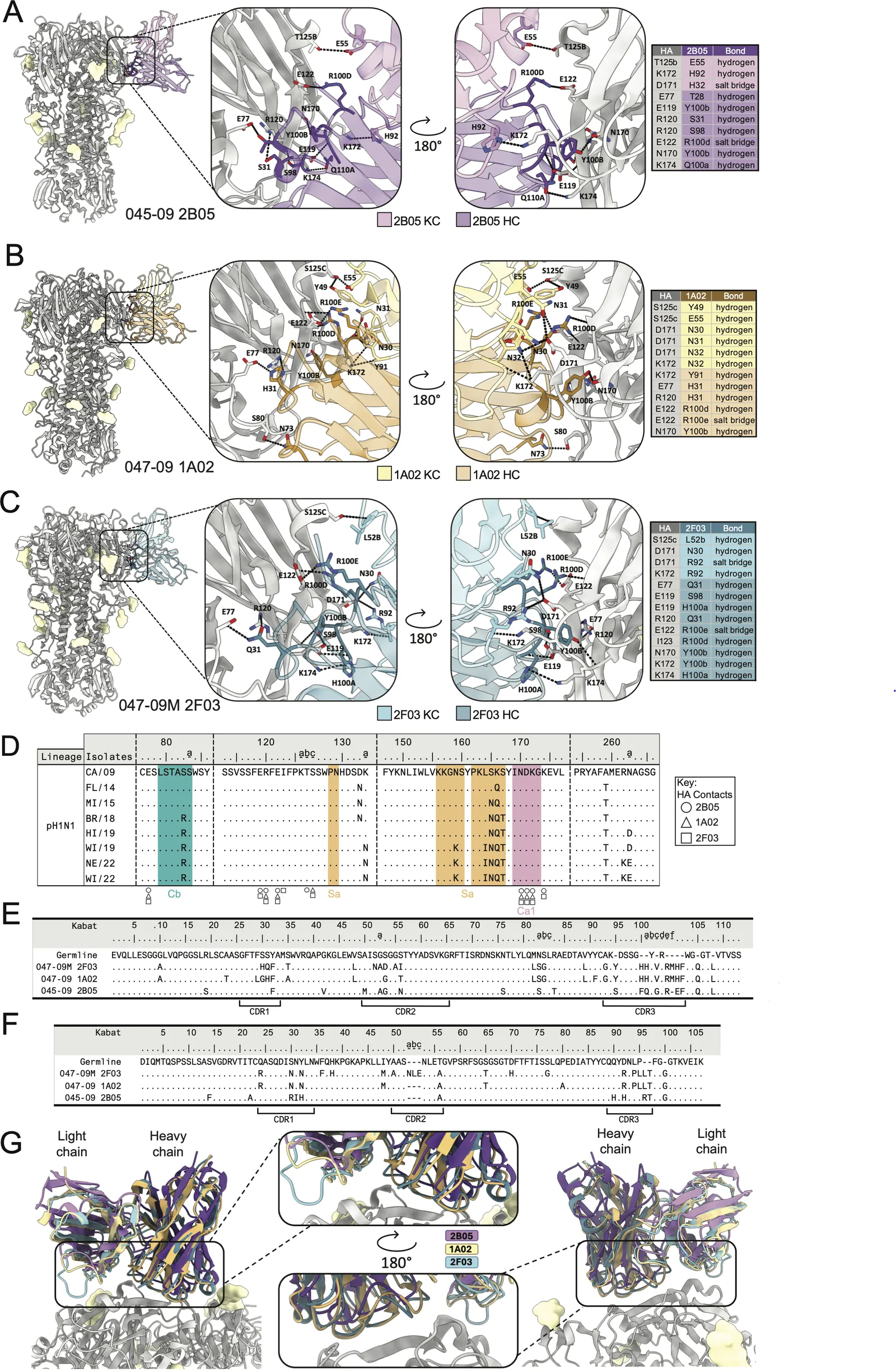
Structures of class 1 lateral patch-specific mAbs. (**A–C**) Cryo-EM structures of 045-09 2B05 (**A**), 047-09 1A02 (**B**), and 047-09M 2F03 (**C**) in complex with A/California/04/2009 HA. Zoomed in are the epitope:paratope interactions. Dashed lines represent contacts between the mAbs and HA. (**D**) Sequence alignment of post-2009 H1N1 viruses with mAb contacts. (**E and F**) Germline sequence alignment of 047-09 1A02, 045-09 2B05, and 047-09M 2F03 by heavy chain (**E**) and light (**F**) chains. (**G**) Overlay of the three class 1 mAbs binding to the HA head in side-to-side view and zoomed-in view of the paratope.

We identified 047-09M 2F03 retained binding to all post-2009 pH1N1 viruses tested (Fig. 2C). 047-09M 2F03 is clonally related to 047-09 1A02 and 047-09 4G02, which lost binding to pH1N1 viruses due to A259T (Fig. 2E). We next sought out to understand if there were structural differences between 047-09M 2F03 and 047-09 1A02 relative to the previously characterized class 1 lateral patch-specific mAb 045-09 2B05 (Protein Data Bank [PDB] 7MEM, Fig. 3A) by determining the cryo-EM structures of 047-09 1A02 (Fig. 3B, Fig. S2 and Table S4) and 047-09M 2F03 (Fig. 3C; Fig. S3, Table S4) in complex with CA/09 HA. Two-dimensional (2D) class averages of the complex exhibited diverse views with visible secondary structures (Fig. S2A and S3A) and three Fabs bound per HA trimer (Fig. S2B and S3B). Iterative classification and refinement in 3D (Fig. S2C and S3C, Table S4) resulted in a final map for 047-09 1A02 and 047-09M 2F03 at 3.7 and 3.06 Å resolutions, respectively (Fig. S2D and S3D, Table S4). The highest resolution features corresponded to the paratope:epitope interface and the interior of the HA head (Fig. S2E and S3E and S4A and B).

All three mAbs analyzed use the same heavy and light chain (V_H_3-23/V_K_1-33) and possess a Y-x-R motif within the HCDR3 that is highly conserved across class 1 lateral patch-binding mAbs (11). Despite their genetic similarities, structures of all three mAbs revealed diverse interactions between each mAb and the lateral patch (Fig. 3A through F). Despite the diverse array of contacts, most HA contacts were in the 120 β-strand and the 170-loop of Ca1, and most contacts were mediated by the heavy chain (Fig. 3A through D). Notably, all three mAbs used a Y-x-R motif in the HCDR3 that contacted residues within the 120 β-strand or the 170-loop, with HA N170 forming a hydrogen bond with Y100b within the HCDR3 of all three mAbs (Fig. 3A through D). Shared HA contacts across all three mAbs included E77 near the major antigenic site Sb, R120 and E122 in the 120 β-strand, and N170 and K172 within the 170-loop (Fig. 3A through D). Despite differences in contacts, all three mAbs have very similar CDR loop positioning, except for the KCDR2 of 047-09M 2F03 (Fig. 3G). The KCDR2 of all three mAbs binds to HA at slightly different positions. 047-09M 2F03 possesses a three-amino acid insertion in the KCDR2, which is likely responsible for the distinct positioning of the KCDR2 (Fig. 3F). 047-09M 2F03 KCDR2 binds to S125c via L52b, whereas 047-09 1A02 contacts S125c via E55 and Y49, and 045-09 2B05 interacts with S125C via E55 (Fig. 3A through C). Together, these structural analyses reveal that despite being from the same clone and similar HA contacts, 047-09M 2F03 and 047-09 1A02 largely use distinct residues to mediate lateral patch specificity.

We identified that A259T was critical for evading both class 1 and 2 lateral patch-specific mAbs (Fig. 2E). However, our structural characterization of class 1 lateral patch mAbs revealed that A259 is neither in direct contact with these antibodies nor is it surface exposed. Rather, A259 lies on a β-strand under the 170-loop of Ca1 that forms a β-sheet with the neighboring 120 β-strand of the lateral patch. To understand the impact of A259T on this epitope and to potentially explain how it mediates antibody evasion, we performed molecular dynamics simulations of CA/09 with A259 or T259 (Fig. S4A and B). Introduction of T259 into CA/09 increased the flexibility of the 170-loop, as well as the β-strand where residue 259 resides (Fig. S5A and B). This predicted increase in flexibility in the 170-loop is likely due to the introduction of the bulkier and polar threonine (T) residue relative to the smaller and non-polar alanine (A) found in the 2009 pH1N1 viruses. The increased flexibility predicted at K174 is likely the reason we observe a decrease in binding affinity for class 1 mAbs (Fig. 2B). Together, these data support that A259T increases the flexibility of K174, which in turn led to antigenic drift. Importantly, this finding provides new biophysical insights into how mutations outside of the epitope can impact recognition by antibodies.

### Class 2 lateral patch-specific mAbs retain binding to pre-2009 H1 HA Isolates

**Fig 4:**
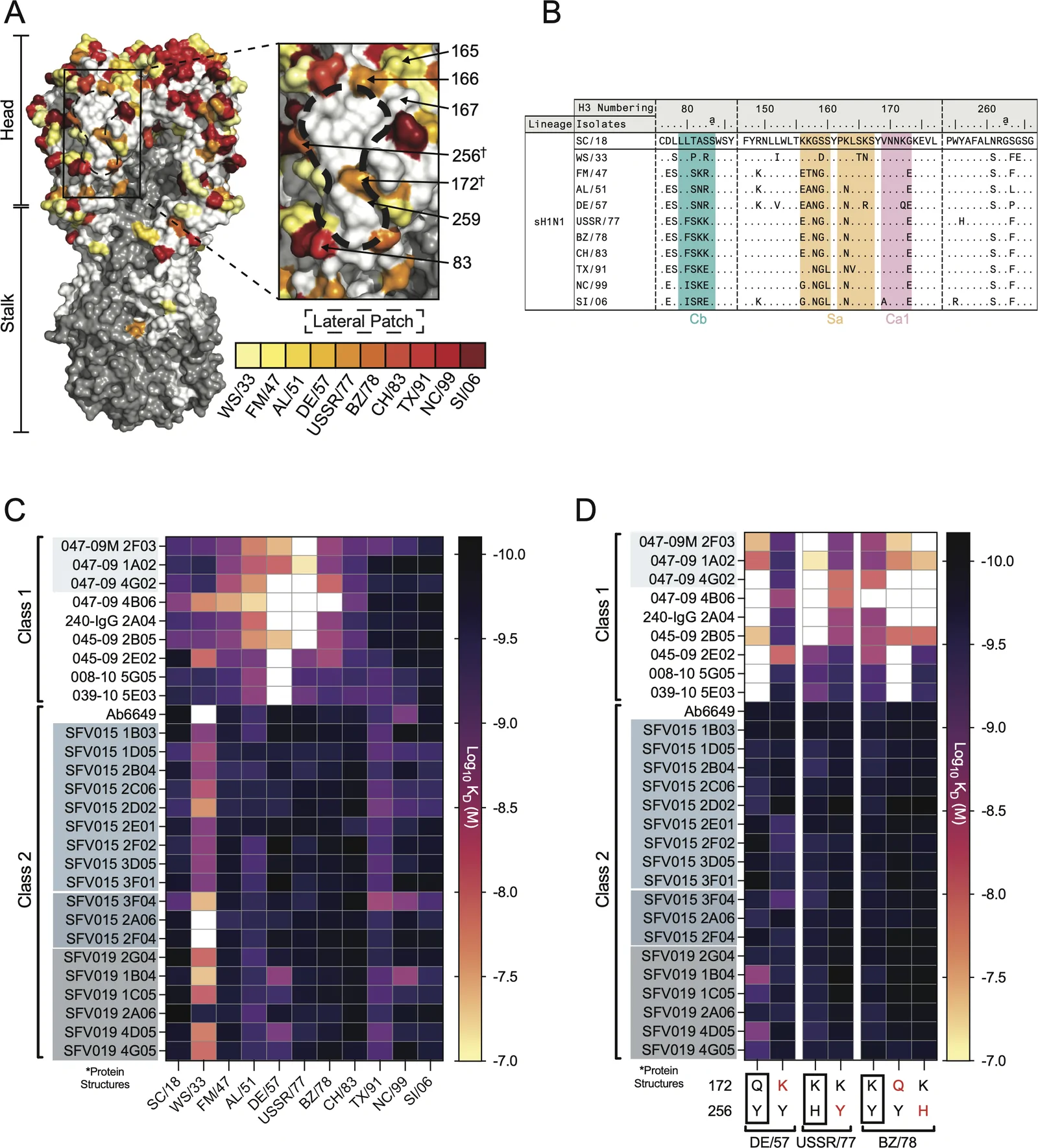
Antigenic structure and binding breadth of mAbs against the lateral patch of pre-2009 sH1N1 viruses. (**A**) Structure of HA from A/South Carolina/1/1918 (PDB: 1RD8), highlighting the lateral patch epitope, and mutations in sH1N1 viruses from 1918 to 2006. The HA1 and HA2 are visualized in white and gray, respectively. (**B**) Amino acid sequence alignment of pre-2009 H1 isolates, focused on residues near or within the lateral patch epitope. Colored shading depicts antigenic sites near the lateral patch. (**C and D**) Apparent affinity of the lateral patch-targeting mAbs screened against pre-2009 sH1N1 isolates (C) and H1 antigens with mutations at positions 172 and 256. mAbs are divided by their class. Clonally related mAbs are highlighted by distinctly shaded blue boxes. White boxes represent no binding.

Next, we tested the conservation and antigenicity of the lateral patch in pre-2009 H1N1 viruses. We observed multiple mutations within and around the lateral patch epitope in sH1N1 viruses collected from 1933 to 2006 (Fig. 4A and B). Class 1 and 2 lateral patch-targeting mAbs cross-reacted with SC/18 and had stronger overall binding affinities than for CA/09 (Fig. S6). Cross-reactivity to SC/18 was not surprising, given that both pandemic isolates are genetically and antigenically related (11, 49–51). However, these antibodies were isolated from donors born in the 1960s–1980s, and their first exposure to H1 would have been an antigenically drifted sH1N1 virus relative to SC/18. We did observe reduced binding affinity of lateral patch-specific mAbs to A/Wilson Smith/1933 (WS/33), the first human influenza isolate collected (52). This virus has been serially passaged through animal hosts and embryonated chicken eggs prior to sequencing (53). As many mutations in WS/33 are absent from prior strains and subsequent drifted variants (Fig. 4B), changes in antigenicity may be due to this serial passaging rather than antigenic drift (42), although we cannot rule out that mutations are not due to antigenic drift, particularly because there is a lack of sequencing data directly before and after 1933. WS/33 has two mutations, S165T and K166N, relative to SC/18 (Fig. 4B), which could impact class 2 mAb binding similar to the K166Q mutation in post-2009 pH1N1 viruses. Consistent with this, WS/33 modestly reduced class 2 mAb binding (Fig. 4C).

Class 1 mAb binding affinity was reduced or completely ablated against influenza isolates collected from 1947 to 1957 and in 1977. Most class 1 mAbs are unable to bind A/Denver/1/1957 (DE/57) and A/USSR/90/1977 (USSR/77), including 047-09M 2F03, which possesses significant breadth against post-2009 viral isolates. Mutation S83R was a recurrent substitution that appeared in four isolates from 1933 to 1957 (Fig. 4D), and this mutation was reacquired in post-2015 pH1N1 isolates (Fig. 2B). Despite this, class 1 mAbs bound WS/33 and A/Fort Monmouth/1/1947 (FM/47) despite the presence of the S83R mutation. However, additional mutations arose in the Cb antigenic site, particularly at residue 82. In FM/47, this strain possessed an A82K mutation. In contrast, A/Albany/12/1951 (AL/51) and DE/57 acquired a K82N mutation, which resulted in a predicted N-glycosylation motif site at position N82. However, position N82 was not maintained in the USSR/77 isolate, as this isolate has K82 (Fig. 4D). Class 1 mAbs had reduced binding to AL/51, compared to FM/47, potentially due to the predicted N-glycosylation site introduced at K82N within the Cb epitope (Fig. 4C). Moreover, the upper portion of the lateral patch also acquired a predicted N-glycosylation motif site at residue 163 in AL/51 (Fig. 4B). Despite this, class 2 mAbs maintained binding to DE/57, suggesting the mutation at position 163, which is found ubiquitously in contemporary sH1N1, does not affect class 2 mAb binding (Fig. 4C). Notably, N163 is not a contact of Ab6649, suggesting why this mutation does not impact mAb binding (13). Class 2 lateral patch mAbs were able to maintain binding to all isolates, including DE/57, despite the mutation K166R (Fig. 4A and C).

Both DE/57 and USSR/77 isolates evaded most class 1 lateral patch mAbs. DE/57 acquired two isolate-specific mutations, K172Q at antigenic site Ca1 and K166R in Sa (Fig. 4B). In contrast, USSR/77 acquired a mutation bordering the lateral patch, Y256H, and a mutation at R83K in the Cb antigenic site (Fig. 4B). However, Q172 in DE/57 was not maintained in USSR/77, and H256 in USSR/77 was not retained in A/Brazil/11/1978 (BZ/78). To test the importance of these two mutations, we reverted the mutations at residues Q172K and H256K in DE/57 and USSR/77, respectively. We then introduced these mutations, K172Q and Y256H, into BZ/78 to test if they were sufficient to ablate mAb binding. Both mutations ablated class 1 mAb binding in BZ/78, whereas DE/57+Q172K and USSR/77+H256Y restored class 1 mAb binding (Fig. 4D). Together, these data show that K172Q and Y256H reduced class 1 mAb binding during the 1950s and 1977. All lateral patch-targeting mAbs bound isolates from 1978 to 2006, likely due to no new mutations in or near the lateral patch during this time frame (Fig. 4A through D). Overall, this demonstrates that both classes of lateral patch-targeting antibodies retained binding to H1 antigens from 1983 to 2006, suggesting the lateral patch remained conserved during this period, despite dramatic evolution in the Cb, Ca1, and Sa major antigenic sites.

The two classes of lateral patch-specific antibodies are from donors with discrete birth years. Class 1 mAbs were from donors born in the mid-1980s, and class 2 mAbs were from donors born in 1961 and 1975 (Table S3). Donors with class 1 antibodies were likely first exposed to sH1N1 viruses from the late 1980s to the early 1990s, which are similar to A/Texas/36/1991 (TX/91). In contrast, donors with class 2 antibodies likely generated their primary responses against sH1N1 after the reemergence of the sH1N1 virus in 1977 (11, 13). Thus, the first H1N1 virus infection may have shaped which antibody class was induced to bind to the lateral patch.

### Lateral patch-targeting mAbs retain binding to enzootic H1

To elucidate the conservation and antigenicity of the lateral patch of enzootic H1 HAs, which pose a risk to human health via potential spillover events, we tested the binding breadth of lateral patch-targeting mAbs to H1 antigens of enzootic origin. These included rHAs from 1A, 1B, and 1C swine lineages of H1N*x* viruses and avian-origin lineage H1.

**Fig 5:**
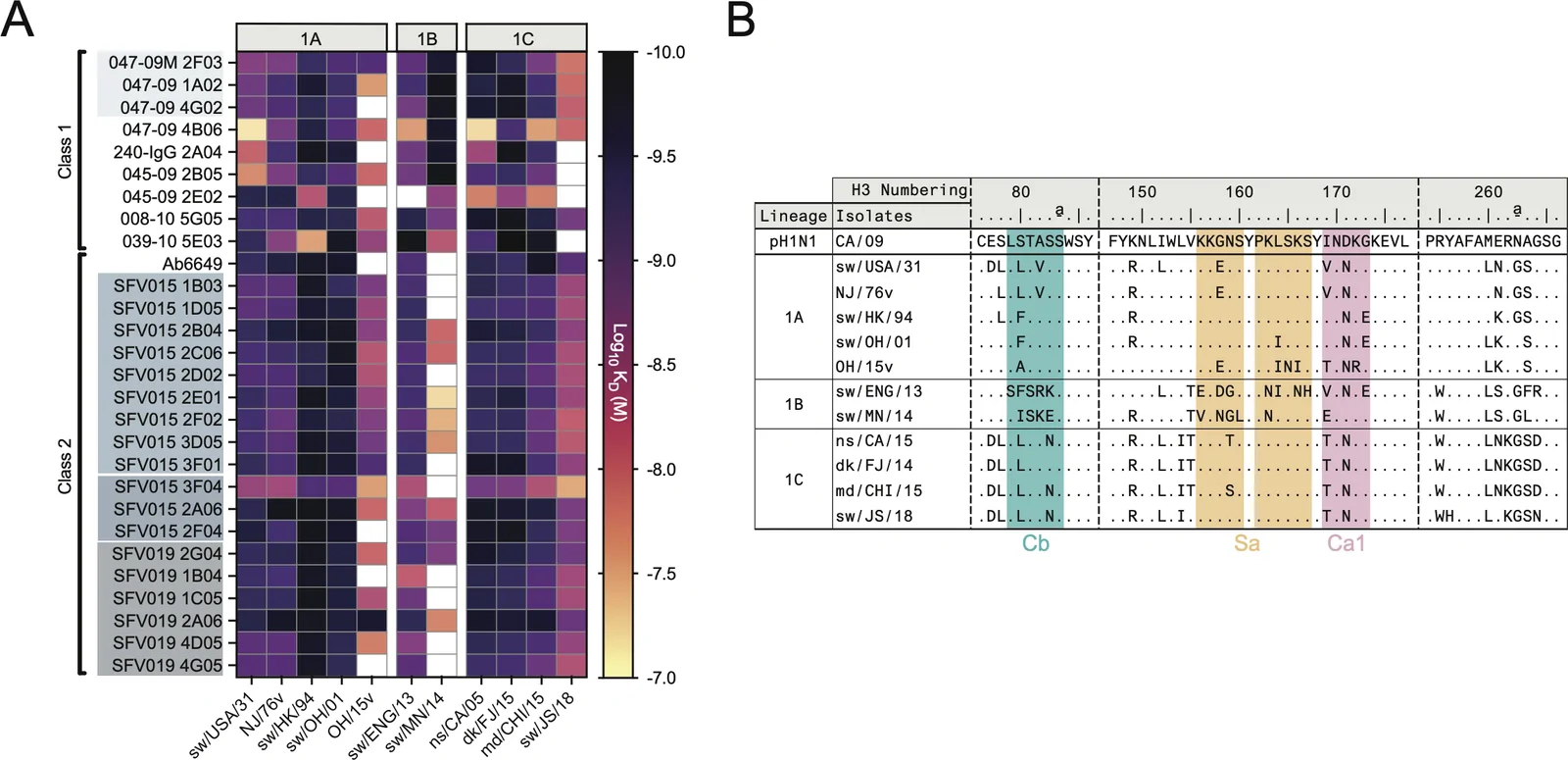
Binding breadth of the lateral patch-targeting mAbs in zoonotic H1 isolates. (**A**) Apparent affinity of the lateral patch-targeting mAbs screened against zoonotic H1N*x* isolates presented in a heat map. mAbs are divided by their class. Clonally related mAbs are highlighted by distinctly shaded blue boxes. White boxes represent no binding. rHAs are grouped by lineages and within chronological order. (**B**) Amino acid sequence alignment of selected zoonotic H1 isolates focused on residues near or within the lateral patch epitope to reference strain CA/09, corresponding to mutations in positions 83, 165-167, and 259. Colored shading depicts antigenic sites near the lateral patch.

We observed that both class 1 and 2 mAbs bound to 1A classical swine lineage viruses from 1931 to 2001 (Fig. 5A), suggesting that this epitope was antigenically conserved during this time period in swine influenza viruses (39, 40). The only exception was the OH/15v isolate, which had reduced binding or completely evaded a subset of class 1 and 2 lateral patch-targeting mAbs (Fig. 5A). The OH/15v isolate was obtained from a lethal human infection with a variant 1A swine H1N1 virus (54). The OH/15v has multiple substitutions in and around the lateral patch, including I166 (Fig. 5B). As K166 is a known contact of the class 2 lateral patch mAb Ab6649 (13), it is plausible that this substitution could be responsible for reduced binding by class 2 lateral patch mAbs. Furthermore, the substitution R172 in the Ca1 antigenic site of OH/15v may have contributed to reduced binding by class 1 mAbs (Fig. 5B). Together, these data support that 1A classical swine viruses have maintained conservation within the lateral patch. However, the evasion of lateral patch-targeting by OH/15v warrants further investigation into whether circulating 1A viruses have undergone antigenic drift that efficiently evades lateral patch-targeting antibodies.

Next, we tested binding of lateral patch-targeting mAbs to two lineage 1B swine isolates. These swine isolates share common ancestors with sH1N1 derived from humans in the 1980s and 1990s. The A/swine/England/063782/2013 (sw/ENG/13) virus is phylogenetically related to A/Chile/1/1983 (CH/83), and A/swine/Minnesota/A01567011/2014 (sw/MN/14) is phylogenetically related to TX/91 (Fig. 1B). Class 2 mAbs maintained binding to sw/ENG/13 but had reduced binding to sw/MN/14 (Fig. 5A). In contrast, class 1 mAbs retained binding to both rHAs, although they have lower affinity to sw/MN/14 relative to sw/ENG/13 (Fig. 5A). Both 1B swine isolates differ in mutations at or near the lateral patch (Fig. 5B). The lack of binding by class 2 mAbs may be due to the substitution E169 in antigenic site Ca1 (Fig. 5B), as this is a binding contact for Ab6649 (13). Although these 1B swine isolates derive from human sH1N1, their genetic and antigenic differences may be the result of divergent antigenic drift in the United States and Europe (30, 41, 55).

Lastly, the lateral patch epitope is well conserved in enzootic isolates of lineage 1C, as all mAbs exhibit high binding affinity to the lateral patch, especially to H1s of avian origin. However, about half of the class 1 mAbs and several class 2 mAbs exhibited reduced binding affinity against the A/swine/Jiangsu/J004/2018 (sw/JS/18) isolate (Fig. 5A). Sw/JS/18 has a Y256H mutation, which is also found in the human isolate USSR/77 (Fig. 5B). Class 1 mAb 047-09M 2F03 was able to bind to this swine isolate, despite not binding to USSR/77 (Fig. 4C). In contrast, lateral patch mAbs 039-10 5E03 and 045-09 2E02 were able to bind to the USSR/77 but were unable to bind to sw/JS/18 (Fig. 4C and 5A). Together, these data support that the lateral patch is well conserved across enzootic H1 viruses, despite evolution at nearby major antigenic sites. Further antigenic characterization of enzootic H1N*x* viruses is necessary to determine whether these viruses are undergoing antigenic drift to evade broadly neutralizing antibodies.

### Antigenic evolution of the lateral patch

Using binding data from our rHA-antibody ELISAs, we assessed the antigenic relatedness of H1 isolates by generating antigenic maps, similar to antigenic cartography. Classically, antigenic cartography maps are generated from hemagglutination inhibition assays with human or ferret sera to visualize antigenic drift over time (43, 56–58). Here, we adapted this approach using lateral patch mAb binding affinities to understand lateral patch antigenicity over time and species, referring to these graphs as antigenic maps.

**Fig 6:**
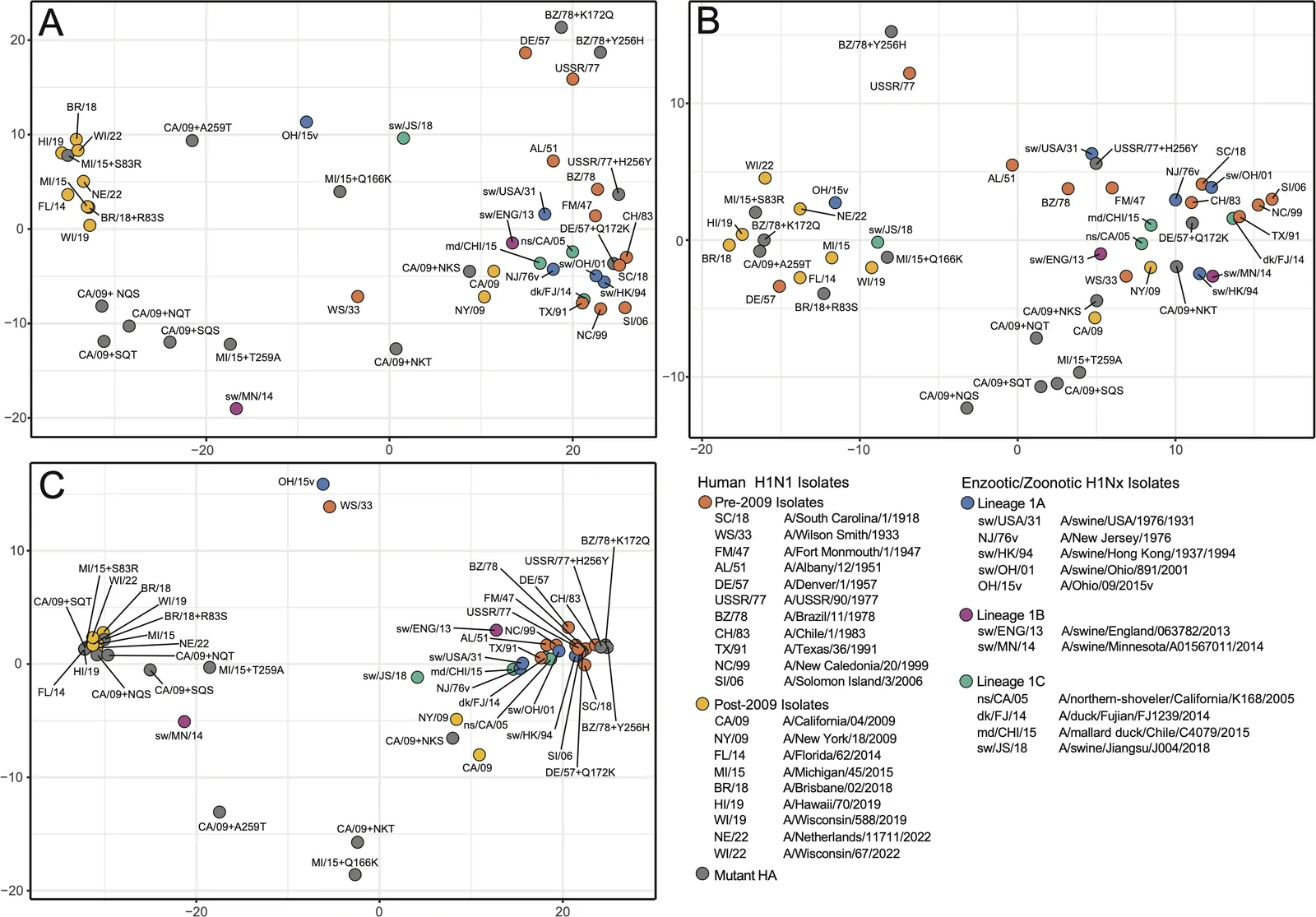
Antigenic relationship of human, enzootic, and mutant rH1s based on lateral patch-specific mAb binding. (**A**) Antigenic map of all lateral patch-targeting mAbs against all H1 antigens. (**B**) Antigenic map of class 1 lateral patch-targeting mAbs. (**C**) Antigenic map of class 2 lateral patch-targeting mAbs.

Analysis of an antigenic map of all lateral patch-targeting mAbs revealed that clustering patterns are largely driven by post-2009 H1N1 strains with K166Q, A259T, and S83R mutations, including mutated post-2009 HAs that possess these mutations (Fig. 6A and B). Furthermore, three distinct sH1N1 isolates (WS/33, DE/57, USSR/77) and the three recently collected swine isolates (sw/OH/15v, sw/MN/14, and sw/JS/18) do not fall into any distinct cluster, which is likely driven by differences in the mAb binding affinity (Fig. 6A).

Next, we generated antigenic maps specifically for class 1 and class 2 mAbs (Fig. 6B and C). HA clustering for class 1 mAbs is largely driven by the presence of A259T and S83R in post-2014 isolates and K172Q and Y256H in sH1N1 viruses (Fig. 6B). An antigenic map of class 2 mAbs revealed tight clusters associated with pre- and post-2009 H1N1 viruses, largely driven by the presence of K166Q. Furthermore, WS/33 harbors mutations at the Sa antigenic sites that likely drive evasion of class 2 lateral patch-targeting mAbs, leading to its separation from other pre-2009 sH1N1 viruses in this map (Fig. 6C).

Lastly, the enzootic isolates demonstrate distinct clustering in all antigenic maps. This likely is reflective of their phylogenetic relatedness. For example, 1A classical swine and the 2009 HA isolates cluster closely with SC/18, and 1B swine viruses cluster near the 1978–2006 sH1N1 antigens (Fig. 1 and 6A through C). However, swine isolates such as OH/15v, sw/MN/14, and sw/JS/18 harbor mutations in and around the lateral patch epitope that largely diminishes binding to class 1 and class 2 lateral patch-targeting mAbs (Fig. 5A, B and 6A through C). Although K166Q in pH1N1 drives the evasion of class 2 antibodies, other substitutions found in Ca1 (E169 and R172), Sa (I166), and Y256H likely contribute to differences in antigenicity relative to human isolates.

## DISCUSSION

In this study, we utilized lateral patch-specific mAbs from humans vaccinated against the 2009 pH1N1 to assess their binding affinity to HA H1 strains from human isolates collected between 1918 and 1957 and between 1977 and 2022 and to enzootic H1N*x* isolates collected from different hosts, lineages, and geographical regions. Most lateral patch-targeting mAbs in our panel bound to human H1 isolates prior to 2014, despite reduced binding affinity against isolates from the 1950s and when sH1N1 reemerged in humans in 1977. Moreover, we found that most lateral patch-targeting mAbs bound to enzootic H1s. Because lateral patch antibodies are demonstrated to be neutralizing against a wide range of H1N1 viruses and mediate protection *in vivo* (11)*,* lateral patch-specific antibodies could mediate protection in the event of an H1N*x* pandemic.

Class 1 lateral patch-targeting mAbs target the lateral patch from a perpendicular angle that prevents overlap with residue 166, which likely preserves binding to K166Q mutant viruses. Class 1 lateral patch mAbs have a Y-x-R motif within the HCDR3 that makes multiple critical contacts with the lateral patch. A prior study revealed that class 1 lateral patch mAbs can drive mutations at positions E119, R120, and A259 (11), with the prior two mutations being contacts of the Y-x-R motif based on structures generated in this study. A more recent study of three clonally related class 1 lateral patch mAbs revealed multiple escape mutations at positions E119, R125, and K172, as well as the introduction of N-glycosylation motifs at positions P122, K123, and E124 (59). All of these sites are critical contacts of the Y-x-R/K motif in class 1 mAbs (11, 59). Despite these potential evolutionary trajectories, only A295T has emerged and persisted in circulating pH1N1 viruses. Moreover, class 2 mAb Ab6649 targets the upper portion of the lateral patch, which overlaps with the Sa antigenic site (18, 19), therefore making this antibody, and likely other class 2 lateral patch-specific mAbs, particularly susceptible to the K166Q mutation found in all post-2014 pH1N1 viruses. Using deep mutational scanning, a study found that substitutions at positions 128, 166, and 171 in pre-2009 sH1N1 can evade Ab6649 and its clonally related member, Ab9207 (60). However, only the mutation at K166Q has been sustained in post-2009 pH1N1 viruses. Overall, these studies highlight how diverse substitutions at different residues in the *in vitro* escape mutants of human H1 HA lateral patch and lateral patch-direct lineages can affect binding affinity and neutralization potency.

Despite the significance of how specific substitutions at different positions affect antibody binding in our data, the broadly reactive H1 lateral patch mAb 047-09M 2F03 bound to all post-2009 H1 HA isolates and many human H1 isolates since 1918. We found that 047-09M 2F03 was evaded only by Y256H found in USSR/77. Y256 is stacked between two arginine residues within the lateral patch epitope. The Y256H mutation could induce a pH-dependent conformational change, as protonation of histidine can result in repulsive interactions with the two arginine residues, thereby increasing overall flexibility within the binding interface. Our data show that K172Q in DE/57 and Y256H in USSR/77 reduced class 1 mAb binding. pH1N1 clade 5a.2a has acquired a K172Q mutation, which could potentially impact class 1 lateral patch antibody binding. As K172 is a contact of class 1 lateral patch mAbs, it is possible that this mutation could be responsible for antigenic drift within the lateral patch of contemporary viruses. mAb 047-09M 2F03 is part of the same clonal family as 047-09 4G02 and 047-09 1A02, though we observed that these latter mAbs do not bind to post-2014 H1N1 viruses due to the A259T mutation. Moreover, other class 1 lateral patch-targeting mAbs, such as 240-IgG 2A04 and 045-09 2B05, retain binding to A259T mutants, but lose binding to HA with S83R. R83 may clash with 045-09 2B05 and 240 IgG 2A04, leading to the reduced binding we observed. It remains unknown what mechanism allows 047-09M 2F03 to retain binding to post-2014 H1N1 viruses. Overall, epitope-specific mAbs are sensitive to distinct lateral patch mutations despite overlapping binding footprints and identical or very similar gene usage.

We identified that the two classes of lateral patch-targeting mAbs were segregated into individuals born in two distinct time periods, with class 1 lateral patch-specific mAbs found in donors born after 1985 and class 2 lateral patch-specific mAbs found in donors born prior to the reemergence of sH1N1 in 1977. In the 1950s, Thomas Francis noted that antibody responses were affected by birth cohort, with certain individuals unable to mount antibody responses to previously circulating H1N1 viruses (61). Whereas class 1 mAbs had decreased or no binding affinity to isolates between 1947 and 1957 and USSR/77. Furthermore, it was previously reported that K166-specific Abs were elicited in individuals born between 1965 and 1979 (22, 23). Donors primed with pre-1985 sH1N1 showed a K166-immunodominant response prior to the acquisition of the N-glycosylation site at position 129 in post-1985 sH1N1 (15). It is noted that N129 is predicted to shield K166 in the hemagglutination-inhibition assay and that this effect was reproducible in an animal study (15). Our data show that class 2 mAbs were from donors born in 1961 and 1975 and are K166-specific mAbs. However, despite the temporally positioned N129 glycan, class 2 mAbs bound to three isolates collected between 1991 and 2006. It is unclear if these mAbs bind to N-linked glycans near the lateral patch or have affinity matured to evade these glycans. Furthermore, our study included only 28 lateral patch-specific mAbs from eight donors, limiting any broad conclusions that can be drawn about these patterns. Thus, further investigation into the repertoire of B cells targeting the lateral patch is warranted, particularly as one class 1 lateral patch mAb, 047-09M 2F03, retains broad binding to recent pH1N1 viruses.

The introduction of IAVs into the human population resulted from zoonotic spillover from swine and avian species. Swine surveillance studies have identified diverse IAV subtypes, clades, and lineages that circulate in distinct regions of the world (62, 63). In addition to the spillover of zoonotic influenza viruses into humans, reverse zoonosis of human influenza viruses back into animals, particularly swine, has created reservoirs of IAVs adapted to humans. Notably, multiple reverse zoonosis events have occurred after the 2009 pH1N1 outbreak, leading to the 1A.3.3.2 lineage in swine worldwide (34, 64, 65). Multiple substitutions at position 166 in the Sa epitope have been observed in swine H1 clades (31, 43, 64, 66). Furthermore, K172R and S83K/R have emerged in the Ca1 and Cb antigenic sites in the Korean 1A.3.3.2 clade (65). Despite this, the rate of amino acid substitution in swine H1N*x* remained low compared to human H1N1 viruses. As antigenic drift occurs more slowly in animal reservoirs due to reduced lifespan and immune pressure on IAVs, this would explain why the lateral patch in enzootic isolates remains conserved.

Studies using age-stratified human sera against swine H1 subtypes have revealed that individuals born in different years retain cross-reactivity against specific subclades in the 1A and 1B lineages (58, 67). This suggests that specific age cohorts can have cross-reactive antibody responses in the event of a spillover. However, Eurasian avian-like H1N*x* viruses remain a concern, as there is minimal cross-reactivity to this lineage, and these viruses continue to evolve across distinct species. Swine 1C viruses, such as sw/JS/18, are known to have pandemic potential, as they have high genetic diversity and show efficient infectivity and aerosol transmission in ferret models, and there are little to no cross-reactive antibodies in human sera against these viruses (68, 69).

Our study supports that the lateral patch was well conserved for nearly 100 years, except for mutations arising in the 1950s and 1977 sH1N1 viruses that evade class 1 antibodies and mutations in post-2014 pH1N1 viruses that evade both class 1 and class 2 mAbs. Conserved sites in HA are often maintained due to functional constraints. However, the lateral patch lacks any described or exhibited function, unlike the conserved receptor binding site (RBS), which mediates binding to sialic acid on the surface of host cells. Understanding the function, or lack thereof, of the lateral patch could reveal why this region has been well conserved across human and enzootic H1N*x* isolates.

As H1N*x* remains in circulation in swine and wild avian species, the recurrence of pandemic-potential H1N*x* virus in humans remains high, as multiple cases of H1N*x* variants have been detected and can cause severe disease (54, 64, 70). However, our data support that lateral patch-targeting mAbs largely retain considerable binding breadth against 1A, 1B, and 1C swine H1N*x* viruses, and memory B cells encoding these antibodies could be harnessed to protect against infection in the event of an outbreak or pandemic, as observed by the 2009 pH1N1 (11). Ongoing surveillance of circulating H1N*x* viruses in enzootic reservoirs can inform the suitability of inducing lateral patch-targeting antibodies by vaccination and the potential of lateral patch-targeting antibodies as prophylactics and therapeutics.

## MATERIALS AND METHODS

### Cell culture

Human embryo kidney (HEK) 293T cells (Thermo Fisher Scientific) were maintained at 37°C with 5% CO_2_ in 15 cm surface-treated tissue culture dishes (Fisher Scientific) in Advanced Dulbecco’s modified Eagle’s medium (DMEM; Gibco) supplemented with 2% ultralow IgG fetal bovine serum (FBS, Gibco), 1% L-glutamine (Gibco), and 1% antibiotic-antimycotic (Gibco). These conditions were maintained throughout the protocol by splitting at approximately 80% confluency.

Expi293F cells (Thermo Fisher Scientific) were maintained at 37°C, 125 rpm, 8% CO_2_ in advanced with 80% humidity in plastic flasks with ventilated caps (Avantor VWR Erlenmeyer Flasks, Sterile, Polycarbonate with 0.22 μm ventilated caps). An OrbiShaker CO_2_ shaker with 19 mm orbitals (Benchmark Scientific) was used for aeration and mixing. These conditions were maintained throughout the protocol. During the maintenance and expansion phase, the cells were split to a concentration of 0.3–0.4 × 10^6^ viable cells/mL when they reached a density of 3–5 × 10^6^ viable cells/mL. This required splitting roughly every fourth day post-subculture. Cell density was determined with a Countess 3 FL Automated Cell Counter (Thermo Fisher Scientific).

### Protein production

#### Monoclonal antibody generation

MAbs used in this study are from other published studies (11, 13, 47, 48). Immunoglobulin heavy- and light-chain plasmids were generated from the previously described study (11). Plasmids for the heavy and light chains of a corresponding mAb, 9 μg each in 2.4 mL of DMEM (Gibco), were mixed with 100 μg of 1 mg/mL of polyethyleneimine (PEI, Kyfora Bio), mixed well by vortexing, and co-transfected into confluent HEK 293T cells for 12–18 h, after which the media were changed to Protein-Free Hybridoma Medium II (PFHM-II, Gibco). The supernatant was harvested and clarified after 5 days. The secreted mAbs were purified from the supernatant by passage over Protein A agarose beads (Pierce), followed by sequential centrifugation and washes with 1 M NaCl and 1× phosphate-buffered saline (PBS). mAbs were eluted using glycine-HCl (0.1 M) and neutralized with Tris-HCl (1 M). Proteins were concentrated and buffer exchanged using Amicon Ultra Centrifugal Filter (Sigma) in 1× PBS. Protein concentration was measured with Nanodrop One C Spectrophotometer (Thermo Scientific).

#### Recombinant HA

Recombinant HA amino acid sequences were obtained from the Global Initiative on Sharing All Influenza Data and cloned into a plasmid with a foldon trimerization domain, AviTag, and 6×HisTag. All constructs possessed a Y98F (H3 numbering) mutation. Nine micrograms of plasmid DNA in 2.4 mL of DMEM (Gibco) was mixed with 100 μg of 1 mg/mL of PEI (Sigma-Aldrich), mixed well by vortexing, and co-transfected into confluent HEK 293T cells for 12–18 h, after which the media were changed to PFHM-II (Gibco). The supernatant was harvested and clarified via centrifugation at the end of the fourth day. Expi293F cells followed a different protocol to prepare a DNA-PEI complex by mixing 100 μg of DNA with 500 μL of PEI in 5 mL of Freestyle 293 Expression Medium (Gibco, per 100 mL of cell culture) and co-transfected into high-density Expi293F cells for 12–18 h, after which the media were replaced with fresh Expi293 expression medium. The supernatant was harvested and clarified via centrifugation at the end of the third day. The secreted HAs were purified from the supernatant using Ni-NTA agarose (QIAGEN), followed by gravity-flow purification with disposable 10 mL columns (Pierce). The supernatant-resin mixture was added at a controlled flow rate and washed with washing buffer (20 mM NaH_2_PO_4_, 500 mM NaCl, and 20 mM imidazole at pH 7.4) and elution buffer (20 mM NaH_2_PO_4_, 500 mM NaCl, and 500 mM imidazole at pH 7.4). The eluate was collected, buffer exchanged, and concentrated using an Amicon Ultra Centrifugal filter (Sigma) with 1× PBS. The protein concentration was measured with bicinchoninic acid protein assays (Pierce) and stored at −80°C until use in experiments.

### Cloning, transformation, and expression of mutant HAs

The mutant recombinant ectodomains of H1 (rHA) were generated using site-directed mutagenesis with mutagenic primers. The combination of mutations found in and around the lateral patch was substituted in rH1 antigens of A/California/04/2009 (CA/09) at the naturally occurring SKS→NQT motif site at positions 165–167 and separately introduced A259T. Mutations were also made at positions 83, 166, and 259 in A/Michigan/45/2015 (MI/15) and 83 in A/Brisbane/02/2018 (BR/18). Furthermore, positions 172 and 256 were selected for mutation in the respective isolates of A/Denver/1/1957 (DE/57), A/USSR/90/1977 (USSR/77), and A/Brazil/11/1978 (BZ/78). All mutations were made by site-directed mutagenesis.

The plasmids were transformed into NEB Alpha-competent *Escherichia coli* cells, following the NEB instruction manual, and 200 μL of each dilution was spread onto a selection plate containing carbenicillin. The plate was incubated at 37°C overnight. The instructions for the QIAGEN Plasmid Plus 96 Miniprep Kit from picked colonies were followed to prepare for DNA samples. Samples were measured for concentration using a NanoDrop One C Spectrophotometer prior to Sanger sequencing (QuintaraBio). The plasmid was maxiprepped using the ZymoPURE II Plasmid Maxiprep Kit, and a glycerol stock was prepared. Constructed DNA plasmids were cloned and transfected into HEK 293T or Expi293F cells, following the methods and materials as described above.

### Antigen-specific ELISAs

Recombinant soluble ectodomain HA was coated on a 96-well high-binding microplate (Corning) at 2 μg/mL in 1× PBS overnight at 4°C. The plates were washed with 1× PBS containing 0.05% Tween-20 and blocked with 1× PBS supplemented with 20% FBS for 1–2 h at 37°C. Antibodies were then serially diluted 1:3 starting at 10 μg/mL and incubated for 1–2 h at 37°C. Horseradish peroxidase-conjugated goat anti-human IgG antibody (diluted 1:1,000; Jackson ImmunoResearch) was used to detect mAb binding, and plates were subsequently developed with Super Aqua Blue ELISA substrate (Invitrogen). Absorbance was measured at 405 nm on a microplate spectrophotometer (BioTek). To standardize the assays, control antibodies with known binding characteristics were included on each plate, and the plates were developed when the control absorbance reached 3.0 ± 0.1 optical density units. All experiments were performed in experimental and technical duplicate. Affinity measurements, as represented by the dissociation constant (Kd) at molar concentrations (M), were calculated in Prism 9 (GraphPad) using non-linear regression analysis.

### MD simulations

As starting structures for our simulations, the available X-ray structure of A/California/04/2009 was used (PDB: 4M4Y). For the A259T mutant, this residue was mutated in MOE using the Protein Builder tool, followed by a local energy minimization. For our simulations, we capped the N- and C-terminal parts of each domain with acetylamide and N-methylamide to avoid perturbations by free charged functional groups. For each HA, we performed three repetitions of 1,000 ns of classical molecular dynamics simulations using the AMBER 24 simulation software package, which contains the pmemd.cuda module. The structures were prepared using CHARMM-GUI. The structure models were placed into cubic water boxes of TIP3P water molecules with a minimum wall distance to the protein of 12 Å, and the charge was neutralized with K^+^ and Cl^−^ ions up to a concentration of 0.15 mM. Parameters for all simulations were derived from the AMBER force field 14SB. Each system was carefully equilibrated using a multistep equilibration protocol. Bonds involving hydrogen atoms were restrained using the SHAKE algorithm, allowing a timestep of 2.0 fs. The systems’ pressure was maintained at 1 bar by applying weak coupling to an external bath using the Berendsen algorithm. The Langevin thermostat was utilized to keep the temperature at 300 K during the simulations.

### MD analysis

For the two investigated HA variants, we calculated the residue-wise B-factor for all frames in the MD simulation as a measure of global flexibility implemented in cpptraj to identify and quantify differences in the conformational diversity between the HA variants. Representative cluster structures of the different HA variants were obtained using the average-linkage hierarchical clustering implemented in cpptraj using a Cα-RMSD distance cutoff of 2 Å. We used PyMOL and ChimeraX (71) to visualize protein structures and electron density maps (PyMOL—The PyMOL Molecular Graphics System, v3.0; Schrödinger, LLC).

### Cryo-EM sample preparation

HA complexes were prepared by mixing Fab with a 3:1 molar ratio of Fab:HA (i.e., 3 Fabs per HA trimer) and incubated for 1 h at room temperature. Octyl-β-glucoside detergent (0.1% wt/vol) was added to the complex to aid in particle tumbling. The final concentration of the sample was 1 mg/mL on the grid. A Vitrobot Mark IV system was used for the preparation of cryoEM grids. The settings were as follows: temperature inside the chamber was 4°C; humidity was 100%; blotting force was 1; wait time was 5 s; and blotting time was varied within a 3.5 and 4 s range. The sample (3 μL) was added to plasma-cleaned 1.2/1.3 copper Quantifoil 300 mesh grid (Electron Microscopy Sciences). The plasma cleaning step was performed in the Solarus 950 plasma system (Gatan) with an Ar/O_2_ gas mix for 25 s. The sample was blotted off for 5 s, and the grids were plunge-frozen into liquid-nitrogen-cooled liquid ethane.

### Cryo-EM data collection, processing, and model building

Cryo grids of A/California/04/2009 with E47K stabilizing mutation in HA2 complexed with FAbs 047-09-1A02 and 047-09M 2F03 were imaged at 190,000× nominal magnification using a Falcon 4i camera on a Glacios microscope at 200 kV, respectively. Automated image collection was performed using EPU from Thermo Fisher Scientific. Images were aligned, dose-weighted, and contrast transfer function (CTF)-corrected in the CryoSPARC Live software platform, with automated image collection also performed using Smart EPU software (Thermo Fisher Scientific). Data processing was carried out in CryoSPARC v4.7.1. Micrographs with CTF fits better than 10.5 Å were selected for further processing, yielding 5,616 micrographs for 047-09M 2F03 and 5,152 micrographs for 047-09-1A02. Initial blob-based particle picking was performed on the first 100 micrographs using a minimum particle diameter of 100 Å and a maximum of 200 Å. These particles were subjected to 2D classification to generate templates, which were then used for template-based picking across all remaining micrographs.

Particles were extracted using a 512-pixel box size and subjected to additional rounds of 2D classification. High-quality 2D class averages from the template-picked particles were used as input for topaz training, enabling the selection of fully occupied HA trimer particles and the generation of an initial 3D reference via ab initio reconstruction. This initial model was further refined through heterogeneous refinement, yielding a single high-quality class that was subsequently refined using non-uniform heterogeneous refinement. Final map resolutions determined by gold-standard Fourier shell correlation, were 3.06 Å for 047-09M 2F03 and 3.93 Å for 047-09-1A02. Initial coordinates for the HA model were taken from the available X-ray structure (PDB accession code: 4M4Y), and the initial antibody structure models for 047-09-1A02 and 047-09M 2F03 were predicted using the tool ImmuneBuilder.

We docked the models into the cryo-EM density map in UCSF ChimeraX. The structure model was built iteratively with COOT followed by real-space refinement in the PHENIX package. The Kabat numbering system (72) was used for 047-09-1A02 and 047-09M 2F03 mAbs and the H3 numbering scheme for HA using the Burke and Smith numbering system (73).

### Structural biology and sequence alignment

A crystal structure was obtained from the Protein Data Bank (PDB) to map out the selected mutations in the structure of the HA from the influenza A/California/04/2009 H1N1 virus (PDB: 4JTV) and A/South Carolina/1/1918 (PDB: 1RD8) with PyMOL. The mutations were mapped using the publicly available Nextstrain data set, focusing on the HA1 H1N1pdm genotype and amino acid evolution from 2009 to 2020, with H3 numbering.

### Phylogenetic analysis

Sequence alignments were manually constructed for the HA H1 amino acid sequences using the MEGA12 platform. The neighbor-joining method was used to infer the amino acid sequence relatedness (74). The percentage of replicate trees in which the associated taxa clustered together in the bootstrap test (1,000 replicates) is shown next to the branches (75). The tree is drawn to scale, with branch lengths in the same units as those of the evolutionary distances used to infer the phylogenetic tree. The evolutionary distances were computed using the Johns-Taylor-Thornton matrix-based method (76) and are in units of the number of amino acid substitutions per site. The rate variation among sites was modeled with a gamma distribution (shape parameter = 5.00). The analytical procedure encompassed 31 amino acid sequences. The pairwise deletion option was applied to all ambiguous positions for each sequence pair, resulting in a final data set comprising 566 positions. Evolutionary analyses were conducted in MEGA12 (77, 78) utilizing up to seven parallel computing threads.

### Antigenic maps

Using antigen-specific ELISA Kd values generated from monoclonal antibody binding to distinct HAs, we constructed a 2D antigenic map using the Racmacs R Library for antigenic cartography (https://acorg.github.io/Racmacs/articles/intro-to-antigenic-cartography.html#converting-a-distance-table-to-a-map). Kd values that were marked as “unstable” and had a value of more than 9_E_-7 were replaced with “9_E_-7” to limit between moderate binding and no binding. Only Kd values less than 9 × 10^−7^ were retained.

## Data Availability

All data to understand the conclusions of this research are available in the main text, supplemental material, or repositories listed below. mAb binding data can be found in the supplemental material. Data are fully available without restriction. 047-09M 2F03 and 047-09 1A02 with the CA/09 model were deposited to the Electron Microscopy Data Bank (EMD-76461 and EMD-76462, respectively) and the Protein Data Bank (12IU and 12IV, respectively).

## SUPPLEMENTAL MATERIAL

Data Set S1 jvi.01265-26-s0001.xlsx All mAb binding data.

Supplemental figures jvi.01265-26-s0002.docx Fig. S1, S5, and S6.

Fig. S2 jvi.01265-26-s0003.docx Cryo-EM of 047-09 1A02.

Fig. S3 jvi.01265-26-s0004.docx Cryo-EM of 047-09M 2F03.

Fig. S4 jvi.01265-26-s0005.docx Density maps of cryo-EM structures.

Supplemental tables jvi.01265-26-s0006.docx Tables S1 to S4.

ASM does not own the copyrights to Supplemental Material that may be linked to, or accessed through, an article. The authors have granted ASM a non-exclusive, world-wide license to publish the Supplemental Material files. Please contact the corresponding author directly for reuse.
