# Supplementary material for: Antigenic and structural analysis of the influenza hemagglutinin lateral patch
Source: J Virol. 2026 Sep 23;100(10):e01265-26. doi: 10.1128/jvi.01265-26 (PMC13712955; doi:10.1128/jvi.01265-26)
Supplement: Supplementary file 1 [file jvi.01265-26-s0002.docx]

**Supplementary Figures**

**Figure S1: ELISA-based binding analyses of MAbs screened against A/California/04/2009.** Binding curves were fitted to determine the apparent binding affinity of class 1 (A-B) and class 2 (C-F) and to assess their clonality.

**Figure S5. Molecular Dynamics analysis of WT HA and A259T variant.** MD analysis of the flexibility of WT (A) and A259T CA/09 (B).

**Figure S6. Affinity measurement of lateral patch-specific mAbs.** (A) Affinity of lateral patch-specific mAbs to SC/18 and CA/09. Lines connect matched mAbs. Data were analyzed using a paired non-parametric Wilcoxon test.
