# Supplementary material for: Antigenic and structural analysis of the influenza hemagglutinin lateral patch
Source: J Virol. 2026 Sep 23;100(10):e01265-26. doi: 10.1128/jvi.01265-26 (PMC13712955; doi:10.1128/jvi.01265-26)
Supplement: Supplementary file 2 [file jvi.01265-26-s0003.docx]

**Figure S2. Cryo-EM data processing and validation of 047-09 1A02 in complex with A/California/04/2009 HA.** (A) Representative micrographs of mAb 047-09-1A02 in complex with A/California/04/2009 HA. Scale bar 80 nm. (B) Representative 2D class averages. Scale bar, 150 Å. (C) Ab-initio and heterogeneous refinements (representative classes shown), Non-Uniform (NU) refinement on the best class with C3 symmetry. (D) The final map is colored based on the local resolution estimation. (E) Angular distribution plot from the final NU refine job and Fourier Shell Correlation of the Electron density map.
