# Supplementary material for: Antigenic and structural analysis of the influenza hemagglutinin lateral patch
Source: J Virol. 2026 Sep 23;100(10):e01265-26. doi: 10.1128/jvi.01265-26 (PMC13712955; doi:10.1128/jvi.01265-26)
Supplement: Supplementary file 4 [file jvi.01265-26-s0005.docx]

**Figure S4. Cryo-EM maps and models of 047-09 1A02 and 047-09M 2F03 bound to A/California/04/2009 HA.** (A-B) Reconstruction densities with docked models of 047-09 1A02 (A) and 047-09M 2F03 (B) bound to A/California/04/2009 HA docked onto corresponding cryo-EM maps.
