# Supplementary material for: Antigenic and structural analysis of the influenza hemagglutinin lateral patch
Source: J Virol. 2026 Sep 23;100(10):e01265-26. doi: 10.1128/jvi.01265-26 (PMC13712955; doi:10.1128/jvi.01265-26)
Supplement: Supplementary file 5 [file jvi.01265-26-s0006.docx]

**Supplementary Tables**

**Table S1. H1 HA influenza virus strains and accession numbers.**

*	rHA generated from all H1 isolates have Y98F to reduce non-specific sialic acid binding

**	sH1N1: seasonal H1N1, derived from 1918 H1N1 virus

***	pH1N1: 2009 pandemic H1N1

†	EPI: Epidemic Preparedness Initiative

‡	Single mutation at HA2 E47K to improve stability

‡‡	n.a.: not applicable

**Table S2. Clade-defining mutations in the H1 HA head domain from the 2010-2020.** List of amino acid mutations based on Nextstrain analysis. Mutations were identified by comparing the post-2009 H1 HA isolates listed in Table S1 with the vaccine-selected reference strain A/California/07/2009.

§E262a and D262a co-circulating in differing clades, 5a.2a.1 and 5a.2a, respectively

†A219 and T219 co-circulating in differing clades, 5a.2a.1 and 5a.2a, respectively

*S186P is a homoplasy, occurring in clades 1, 5b, 6, 6b.1 and 6b.1a, before occurring in clade 5

‡Q192E occurred in an earlier clade (5a.1)

^a^Mutations found in the Ca1 antigenic sites

^b^Mutations found in the Ca2 antigenic sites

^c^Mutations found in the Cb antigenic sites

^d^Mutations found in the Sa antigenic sites

^e^Mutations found in the Sb antigenic sites

**Table S3: MAbs identification, gene usage, and origin.**

| **Map** | **047-09M**  **2F03 Fab  + H1 HA (Cal09+E47K)** | **047-09**  **1A02 Fab  + H1 HA (Cal09+E47K)** |
| --- | --- | --- |
| EMDB | 76461 | 76462 |
| PDB | 12IU | 12IV |
| **Data collection** |  |  |
| Microscope | TFS Glacios | TFS Glacios |
| Voltage (kV) | 200 | 200 |
| Detector | Falcon 4 | Falcon 4 |
| Recording mode | Counting | Counting |
| Nominal magnification | 190,000x | 190,000x |
| **EM data processing** |  |  |
| Number of movie micrographs | 5616 | 5082 |
| Number of particle images in map | 135,824 | 87,530 |
| Symmetry | C1 | C3 |
| Map resolution (FSC 0.143; Å) | 3.06 | 3.7 |
| **Structure building and validation** |  |  |
| Number of atoms in deposited model | 17,160 | 16,831 |
| Residues | 2154 | 2124 |
| Glycans | 18 | 14 |
| MolProbity score | 1.45 | 1.32 |
| Clashscore | 7.05 | 5.43 |
| Map correlation coefficient, CC (Mask) | 0.79 | 0.74 |
| d FSC model (0.5; Å) | 3.7 | 4.1 |
| RMSD Bonds |  |  |
| Bond length [Å] | 0.005 | 0.006 |
| Bond angles [˚] | 0.966 | 1.368 |
| Ramachandran plot |  |  |
| Favored (%) | 97.75 | 97.90 |
| Allowed (%) | 2.11 | 2.10 |
| Outliers (%) | 0.14 | 0.00 |
| Side chain rotamer outliers (%) | 0 | 0 |
| Cβ outliers (%) | 0 | 0 |

**Table S4: Cryo-EM data collections, refinement, and validation statistics**
